# Supplementary material for: Production of a monolithic fuel cell stack with high power density
Source: Nat Commun. 2022 Mar 10;13:1263. doi: 10.1038/s41467-022-28970-w (PMC8913829; doi:10.1038/s41467-022-28970-w)
Supplement: Supplementary file 1 — Supplementary information [file 41467_2022_28970_MOESM1_ESM.pdf]

Supplementary Information

Production of a monolithic fuel cell stack with high power density

Stéven Pirou*, Belma Talic, Karen Brodersen, Anne Hauch, Henrik Lund Frandsen, Theis Løye Skafte, Åsa H. Persson, Jens V. T. Høgh, Henrik Henriksen, Maria Navasa, Xing-Yuan Miao, Xanthi Georgolamprou, Søren P. V. Foghmoes, Peter Vang Hendriksen, Eva Ravn Nielsen, Jimmi Nielsen, Anders C. Wulff, Søren H. Jensen, Philipp Zielke, Anke Hagen*

Affiliations

Department of Energy Conversion and Storage, Technical University of Denmark, Kgs. Lyngby, Denmark

Stéven Pirou, Belma Talic, Karen Brodersen, Anne Hauch, Henrik Lund Frandsen, Theis Løye Skafte, Åsa H. Persson, Jens V. T. Høgh, Henrik Henriksen, Maria Navasa, Xing-Yuan Miao, Xanthi Georgolamprou, Søren P. V. Foghmoes, Peter Vang Hendriksen, Eva Ravn Nielsen, Jimmi Nielsen, Anders C. Wulff, Søren H. Jensen, Philipp Zielke & Anke Hagen

* corresponding author

e-mail address corresponding authors: stepir@dtu.dk, anke@dtu.dk

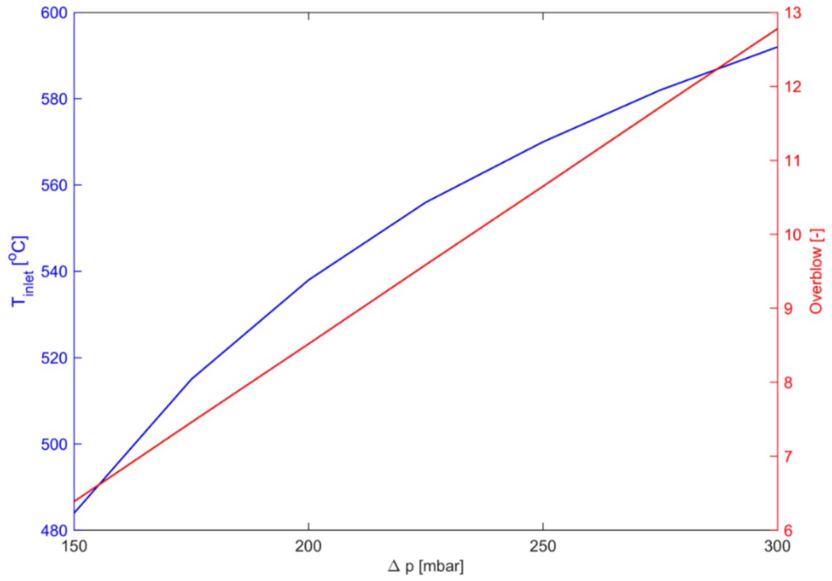

Supplementary Figure 1 | Results of calculation of the air flow (overblowing) for cooling the monolith stack with a footprint of 4 × 7 cm^2 (3 × 6 cm^2 of active area). A 3D multiphysics model built in COMSOL Multiphysics was used to study the size of the gas channels associated with the cooling of the monolith stack. In this model, the overall conservation laws are solved on a homogenised volume, and the microstructural details are introduced through effective material parameters. The model includes current, heat, and mass transport, including gas flows in channels, and finally, an assessment of the mechanical stresses in the structure. Here, the calculated relation between the gas overblow, pressure drop, and inlet gas temperature, which ensures that the stack temperature never exceeds 700 °C, is reproduced when the stack is operated at 0.67 A/cm^2. The calculations confirm that cooling of the monolith stack is feasible with achievable gas channel dimensions (250 × 500 μm^2) and a maximum pressure drop of 300 mbar.

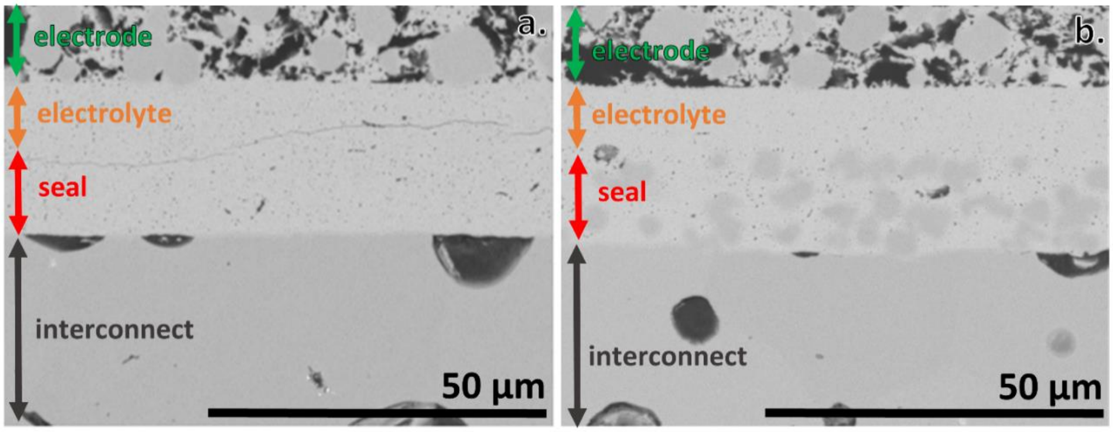

Supplementary Figure 2 | SEM images of fractured and polished cross-sections of two monolithic SOFCs prepared with different seal compositions. Monolithic fuel cell prepared using (a) purely ceramic seals and (b) FeCr/ScYSZ composite seals. A crack-free device could be obtained only when the seal was prepared using the composite.

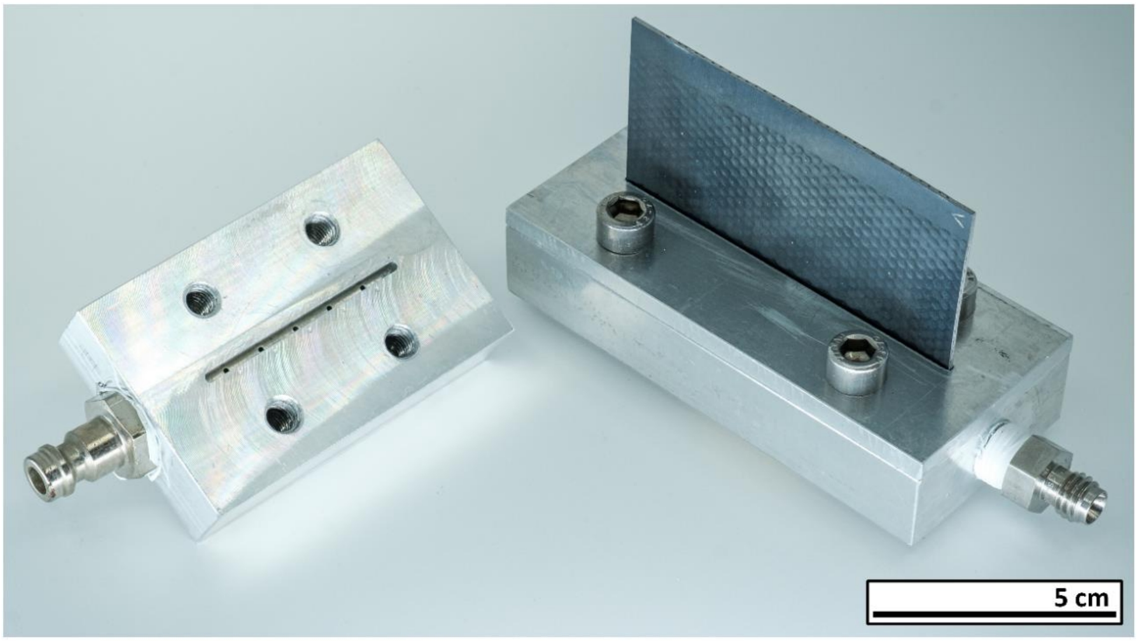

Supplementary Figure 3 | Photograph of the clamping fixture for infiltration. The monolith is placed in a slit and held in place by screws. Leakage is avoided by sealing the outer edge of the monolith with rubber.

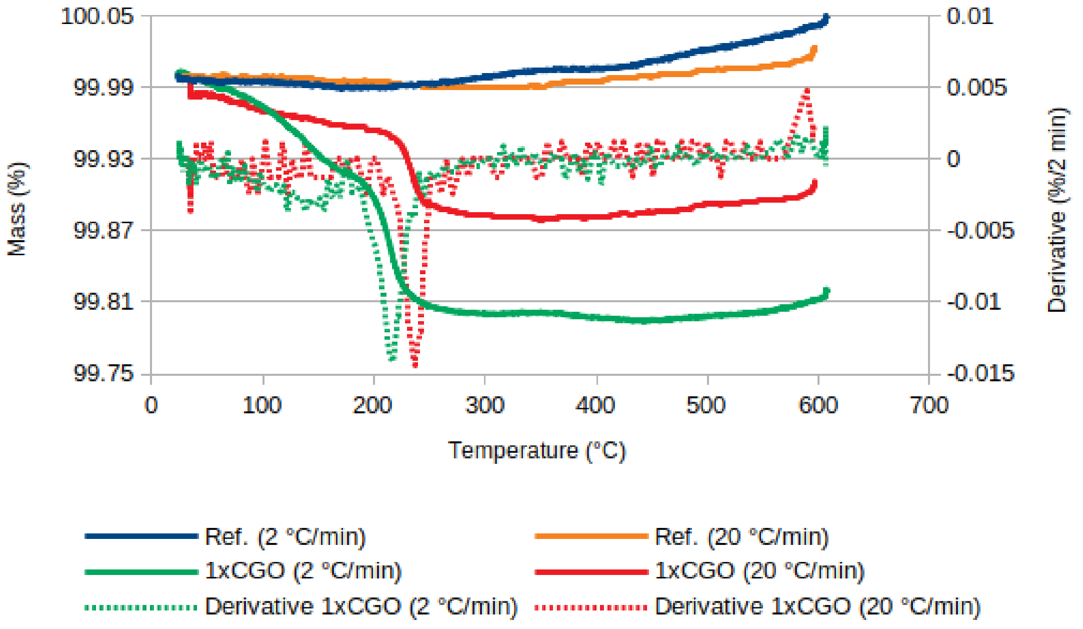

Supplementary Figure 4 | Thermogravimetric analysis of 4 samples heated to 600 °C at either 2 °C/min or 20 °C/min. Two reference samples did not show any weight loss, but rather a weight gain due to steel oxidation. The weight losses of the infiltrated samples appear to be complete at around 300 °C, after which oxidation begins. A temperature of 325 °C was chosen in this study to enable complete decomposition of the precursors whilst minimising unintended corrosion.

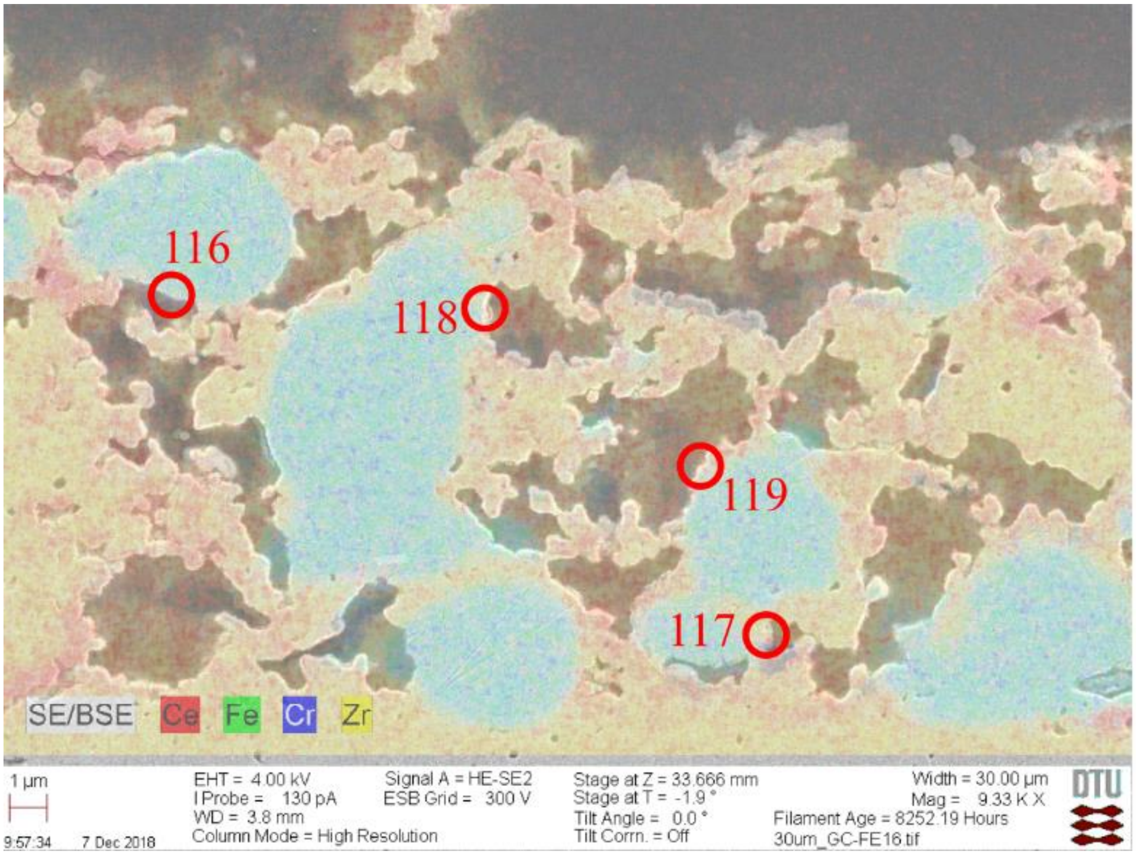

Supplementary Figure 5 | Energy dispersive spectroscopy (EDS) analysis of the infiltrated monolith electrode near a gas channel. The cell was infiltrated with 3 cycles. The red circles indicate the points where quantitative EDS analysis was conducted. All the four points showed nonzero atomic percentages for Ce, indicating the presence of CGO. See Supplementary Table 1 for further information.

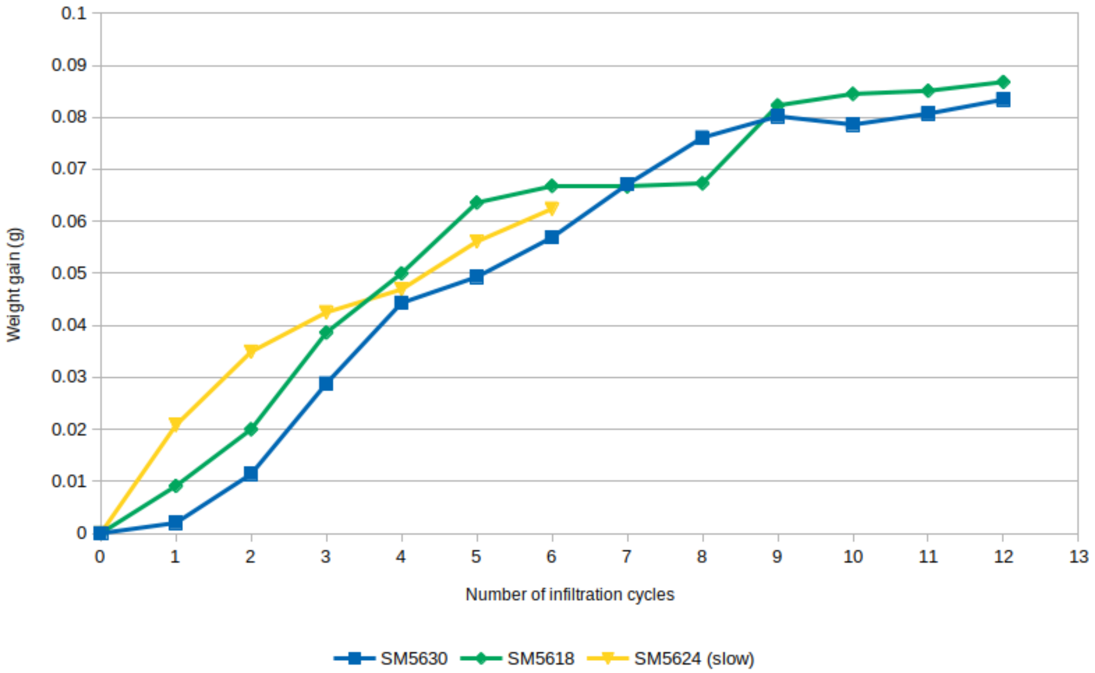

Supplementary Figure 6 | Weight gain as a function of infiltration cycles for three different monoliths.

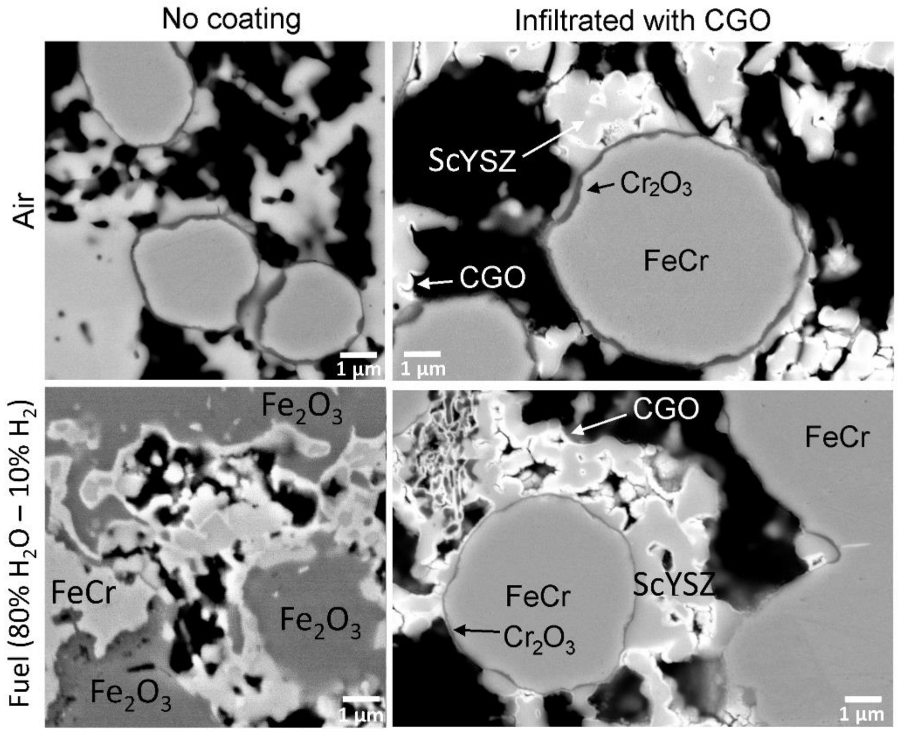

Supplementary Figure 7 | Cross-sectional SEM images of the SRU monoliths after oxidation in air or 80 % H_2O–20 % H_2 for 100 h at 650 °C.

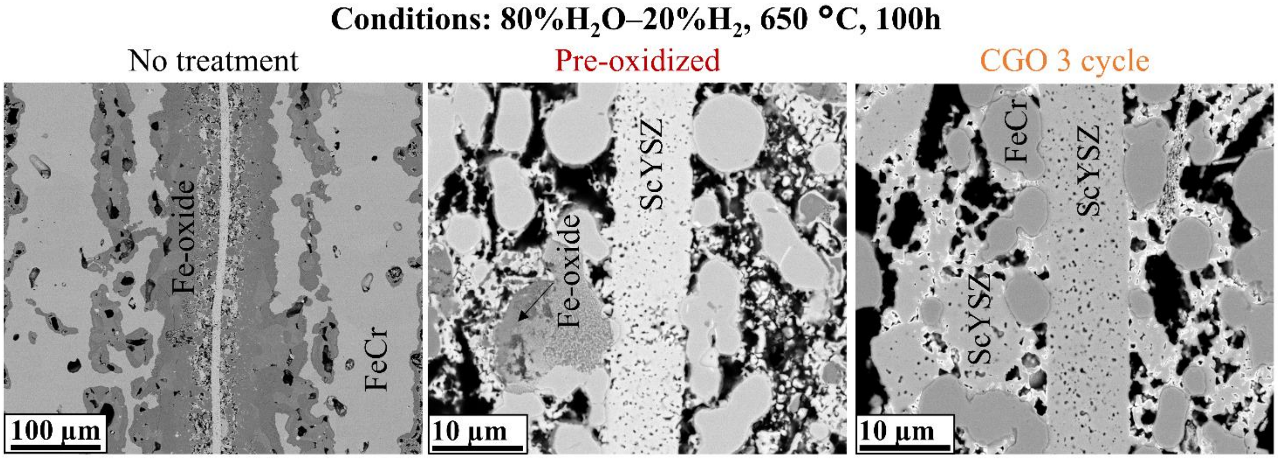

Supplementary Figure 8 | Cross-sectional SEM images of the monoliths after heat treatment at 650 °C in 80% H_2O–20% H_2 for 100 h with and without corrosion protective treatment. Note the scale difference. The corrosion experiments were conducted on non-optimised monoliths (leaky electrolyte). Corrosion stability is greatly enhanced by the short pre-oxidation treatment which leads to the formation of a protective chromia scale, considerably mitigating oxidation of Fe (compare the pictures on the left and the centre). Combining a CGO infiltration and a pre-oxidation treatment further improves the corrosion resistance (compare the pictures on the right and the centre) – as evidenced by the lack of Fe-oxides after the combined treatment (picture on the right).

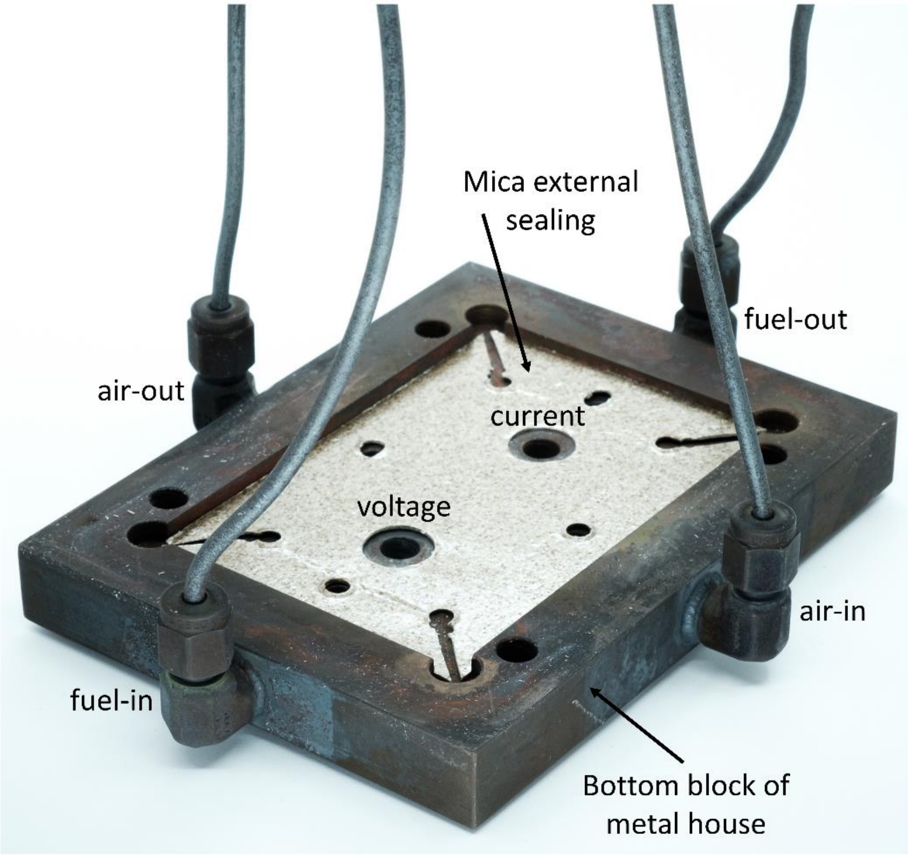

Supplementary Figure 9 | Photograph of the opened test house showing the fuel inlet/outlet, air inlet/outlet, as well as positions of voltage and current probes.

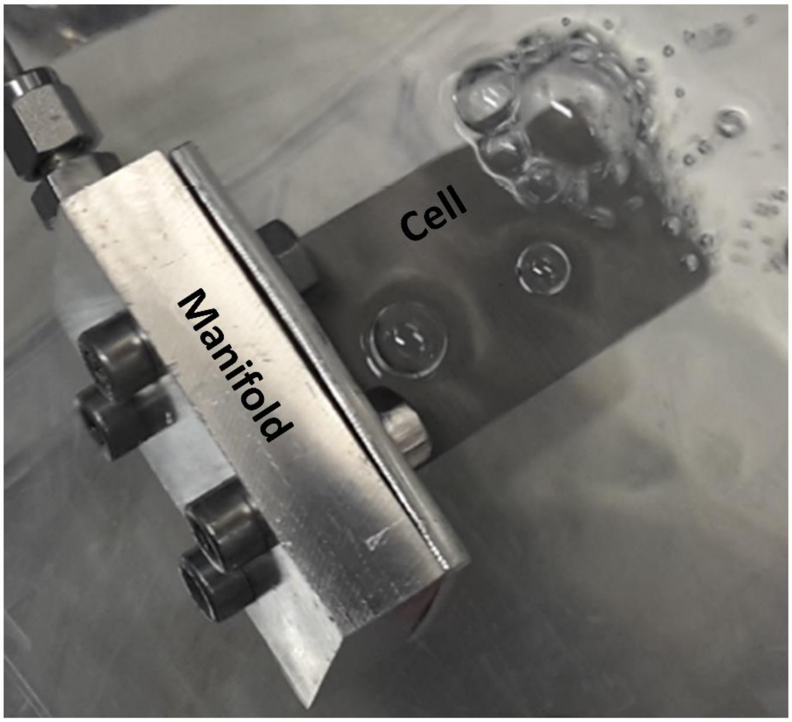

Supplementary Figure 10 | Photograph of the leak-test set-up developed in-house. N_2 is delivered to the manifold using a mass flow controller. The monolith is placed in the slit of the manifold and held in place by screws. The gas is fed through the gas distribution channels. Leakage is avoided by sealing the outer edge of the monolith with rubber. Pin-holes in the interconnect layers can easily be detected by bubbles as the monolith is submerged in water.

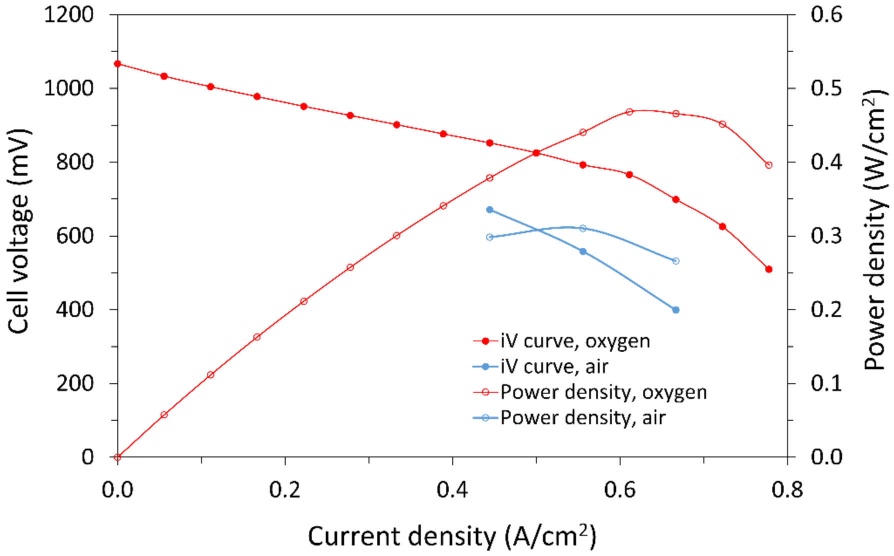

Supplementary Figure 11 | Electrochemical performance of SRU. i–V curve and power density of SRU monolith cell with an active cell area of ~18 cm^2 measured at 780 °C, dry H_2 (25 l/h) to the fuel electrode, and air or O_2 to the oxygen electrode (100 l/h).

Supplementary Table 1 | Atomic percentages obtained from point EDS analysis in Supplementary Figure 5.

Area	Sc	Cr	Fe	Zr	Ce	Gd
116	0.31	23.08	68.42	6.43	0.77	0.00
117	0.03	17.56	56.05	20.55	1.81	0.00
118	0.74	21.05	68.79	7.27	1.03	0.12
119	0.93	19.07	54.37	20.06	1.58	0.00
